# Supplementary material for: Human cytomegalovirus interactome analysis identifies degradation hubs, domain associations and viral protein functions
Source: eLife. 2019 Dec 24;8:e49894. doi: 10.7554/eLife.49894 (PMC6959991; doi:10.7554/eLife.49894)
Supplement: Transparent reporting form [file elife-49894-transrepform.pdf]

eLIFE
1st Floor
24 Hills Road
Cambridge CB2 1JP, UK
P 01223 855340
W elifesciences.org
T @elife

eLife's transparent reporting form

We encourage authors to provide detailed information *within their submission* to facilitate the interpretation and replication of experiments. Authors can upload supporting documentation to indicate the use of appropriate reporting guidelines for health-related research (see EQUATOR Network), life science research (see the BioSharing Information Resource), or the ARRIVE guidelines for reporting work involving animal research. Where applicable, authors should refer to any relevant reporting standards documents in this form.

If you have any questions, please consult our Journal Policies and/or contact us: editorial@elifesciences.org.

Sample-size estimation

- You should state whether an appropriate sample size was computed when the study was being designed
- You should state the statistical method of sample size computation and any required assumptions
- If no explicit power analysis was used, you should describe how you decided what sample (replicate) size (number) to use

Please outline where this information can be found within the submission (e.g., sections or figure legends), or explain why this information doesn't apply to your submission:

Sample-size estimation does not directly apply to the present submission. Two types of sample size could be considered to apply to this data. (a) The total number of viral genes (n=172), and negative controls that were included to assist elimination of background non-specific interactions and estimation of false discovery rates. The minimum number of genes was based on prior studies using similar analysis pipelines (for example, Sowa, Bennett, Gygi, & Harper, 2009). See the legend for **Figure 1** and '**Interactor identification with CompPASS**' section of the Methods.
(b) The number of technical replicates (n=2) for mass spectrometry was chosen firstly based on prior studies using the same analysis pipeline (for example, Huttlin et al Cell 2015; Huttlin et al Nature 2017) and secondly in order to allow infection of all samples with the same pool of virus. Information about this type of sample size can be found in the results section under the header '**Construction of the HCMV-host interactome'** and the legend of **Figure 1**. Further details of how the samples were generated can be found in the Materials and Methods section.

Replicates

- You should report how often each experiment was performed
- You should include a definition of biological versus technical replication
- The data obtained should be provided and sufficient information should be provided to indicate the number of independent biological and/or technical replicates
- If you encountered any outliers, you should describe how these were handled
- Criteria for exclusion/inclusion of data should be clearly stated

- High-throughput sequence data should be uploaded before submission, with a private link for reviewers provided (these are available from both GEO and ArrayExpress)

Please outline where this information can be found within the submission (e.g., sections or figure legends), or explain why this information doesn't apply to your submission:

For the IP-MS dataset, samples were analysed in technical duplicate, as described for this type of approach in Huttlin et al Cell 2015 and Huttlin et al Nature 2017. Specifically, for each viral bait, stable cell lines were seeded in biological duplicate for culture, infection, lysis and immunoprecipitation. Duplicate lysates were then combined and processed further, yielding two technical duplicates of the same biological material. These were analysed by LC-MS/MS separately. Information about replicates can be found in the figure legend of **Figure 1**. Further details of how the samples were generated can be found in the Materials and Methods section.

No samples or data were excluded from analysis. As described in the legend to **Figure 1** and Methods section ('**Interactor identification with CompPASS**' section), interaction data were filtered to identify high confidence interacting proteins (HCIPs) and very high confidence interacting proteins (VHCIPs) using CompPass and CompPass plus software.

Statistical reporting

- Statistical analysis methods should be described and justified
- Raw data should be presented in figures whenever informative to do so (typically when N per group is less than 10)
- For each experiment, you should identify the statistical tests used, exact values of N, definitions of center, methods of multiple test correction, and dispersion and precision measures (e.g., mean, median, SD, SEM, confidence intervals; and, for the major substantive results, a measure of effect size (e.g., Pearson's r, Cohen's d)
- Report exact p-values wherever possible alongside the summary statistics and 95% confidence intervals. These should be reported for all key questions and not only when the p-value is less than 0.05.

Please outline where this information can be found within the submission (e.g., sections or figure legends), or explain why this information doesn't apply to your submission:

All information reporting statistics can be found in the relevant figure and table legends. Exact p-values are reported in the relevant supplementary or source data files. Additionally, a summary of statistics in this manuscript can be found in the '**Statistical analysis**' section of the Methods.

(For large datasets, or papers with a very large number of statistical tests, you may upload a single table file with tests, Ns, etc., with reference to sections in the manuscript.)

Group allocation

- Indicate how samples were allocated into experimental groups (in the case of clinical studies, please specify allocation to treatment method); if randomization was used, please also state if restricted randomization was applied
- Indicate if masking was used during group allocation, data collection and/or data analysis

Please outline where this information can be found within the submission (e.g., sections or figure legends), or explain why this information doesn't apply to your submission:

For the IP-MS analyses, samples were distributed into two experimental groups according to the number of predicted transmembrane domains in the viral bait. Baits with ≤1 transmembrane domain (n=153) were solubilized using a buffer containing the detergent NP-40. Baits predicted to have two to eight transmembrane domains (n=18) were solubilized using a buffer containing Digitonin. Masking was not appropriate during group allocation, data collection or data analysis for these types of sample. Further details of this approach can be found in the legend to **Figure 1** and in '**IP and protein digestion for proteomic experiments**' in the Methods.

Additional data files ("source data")

- We encourage you to upload relevant additional data files, such as numerical data that are represented as a graph in a figure, or as a summary table
- Where provided, these should be in the most useful format, and they can be uploaded as "Source data" files linked to a main figure or table
- Include model definition files including the full list of parameters used
- Include code used for data analysis (e.g., R, MatLab)
- Avoid stating that data files are "available upon request"

Please indicate the figures or tables for which source data files have been provided:

eLife Sciences Publications, Ltd is a limited liability non-profit non-stock corporation incorporated in the State of Delaware, USA, with company number 5030732, and is registered in the UK with company number FC030576 and branch number BR015634 at the address 1st Floor, 24 Hills Road, Cambridge CB2 1JP | August 2014

Figure 2 and ‘Figure 2 – Figure Supplement 1’ - Source data can be found in Supplemental File 4A-B
Figure 3D, 4B, 5A, 5G, 6D, ‘Figure 4-Figure Supplement 1’, ‘Figure 6-Figure Supplement 1A’ - Source data can be found in Supplemental File 2B
Figure 4A – Source data can be found in Supplemental File 5
Figure 5B, 5F – from Nightingale et al, Cell Host & Microbe 2018 (PMID 30122656)
Figure 5C - from Nightingale et al, Cell Host & Microbe 2018 (PMID 30122656) and Stern-Ginossar et al, Science 2012 (PMID 23180859)
Figure 6B, ‘Figure 1 - Figure Supplement 1E’ – from Weekes et al, Cell 2014 (PMID 24906157)
Figure 6C I – Source data can be found in Supplemental File 7A
Figure 6C II – Source data can be found in Supplemental File 7B
Figure 6F – Source data can be found in the file ‘Figure 6F - source data 1’
Figure 6G – Source data can be found in the file ‘Figure 6G - source data 1’
Figure 7A – Source data can be found in Supplemental File 7C
Figure 7B - Source data can be found in Supplemental File 7D
‘Figure 1 – Figure Supplement 1A’ - Source data can be found in Supplemental File 1A
‘Figure 1 – Figure Supplement 1B’ - Source data can be found in Supplemental File 1B
‘Figure 1 – Figure Supplement 1C’ - Source data can be found in the file ‘Figure 1 – Figure Supplement 1C - source data 1’
‘Figure 1 – Figure Supplement 1D’ - Source data can be found in the file ‘Figure 1 – Figure Supplement 1D - source data 1’
‘Figure 1 – Figure Supplement 2A’ – Source data can be found in Supplementary File 2A
‘Figure 1 – Figure Supplement 2B’ – Source data can be found in Supplementary File 3
‘Figure 2 – Figure Supplement 2A’ – Source data can be found in Supplementary File 4C
‘Figure 2 – Figure Supplement 2B’ – Source data can be found in Supplementary File 4D

eLife Sciences Publications, Ltd is a limited liability non-profit non-stock corporation incorporated in the State of Delaware, USA, with company number 5030732, and is registered in the UK with company number FC030576 and branch number BR015634 at the address 1st Floor, 24 Hills Road, Cambridge CB2 1JP | August 2014
